# Is there a bilingual advantage in the ANT task? Evidence from children

**DOI:** 10.3389/fpsyg.2014.00398

**Published:** 2014-05-07

**Authors:** Eneko Antón, Jon A. Duñabeitia, Adelina Estévez, Juan A. Hernández, Alejandro Castillo, Luis J. Fuentes, Douglas J. Davidson, Manuel Carreiras

**Affiliations:** ^1^Basque Center on Cognition, Brain and LanguageDonostia-San Sebastian, Spain; ^2^Departamento de Psicología Cognitiva, Social y Organizacional, Faculty of Psychology, University of La LagunaLa Laguna, Spain; ^3^Departamento de Psicobiología y Metodología de las Ciencias del Comportamiento, Faculty of Psychology, University of La LagunaLa Laguna, Spain; ^4^Departamento de Psicología Básica y Metodología, Faculty of Psychology, University of MurciaMurcia, Spain; ^5^University of GranadaGranada, Spain; ^6^Ikerbasque, Basque Foundation for ScienceBilbao, Spain; ^7^Departamento de Lengua Vasca y Comunicación, University of the Basque Country EHU/UPVBilbao, Spain

**Keywords:** bilingual advantage, inhibitory skills, executive control, attention, ANT task

## Abstract

Bilinguals have been shown to outperform monolinguals in a variety of tasks that do not tap into linguistic processes. The origin of this bilingual advantage has been questioned in recent years. While some authors argue that the reason behind this apparent advantage is bilinguals' enhanced executive functioning, inhibitory skills and/or monitoring abilities, other authors suggest that the locus of these differences between bilinguals and monolinguals may lie in uncontrolled factors or incorrectly matched samples. In the current study we tested a group of 180 bilingual children and a group of 180 carefully matched monolinguals in a child-friendly version of the ANT task. Following recent evidence from similar studies with children, our results showed no bilingual advantage at all, given that the performance of the two groups in the task and the indices associated with the individual attention networks were highly similar and statistically indistinguishable.

## Introduction

The so-called “bilingual advantage” (Kroll and Bialystok, 2013), broadly understood as enhanced executive cognitive control for bilinguals as compared to monolinguals, has attracted very much interest in recent years. Different hypotheses have been proposed to account for this bilingual advantage, all of which predict that bilingual individuals will perform better than their monolingual peers in processing incongruent or salient irrelevant information. While there has been considerable evidence to date supporting a bilingual advantage, very recently there has also been an increase in the number of studies showing a similar performance of bilinguals and monolinguals in non-linguistic executive control tasks. The present study provides data collected from a large sample of carefully matched bilinguals and monolinguals suggesting that the so-called bilingual advantage is not generalizable and replicable when the samples are properly controlled.

One of the most commonly studied tasks in which bilinguals have been claimed to outperform monolinguals is the classic Stroop task (Stroop, 1935). In this task, participants have to name the color in which target words are printed. The difference between the latencies to incongruent trials (i.e., the target word to be named is the name of a color and is printed in a different ink color; e.g., the word “green” printed in red color) and the latencies to congruent trials (i.e., target word and its color match; e.g., the word “green” printed in green) is the Stroop effect, and is an index of inhibitory control. The Stroop effect was found to be smaller in bilingual participants than in their monolingual peers and this difference has been claimed to be especially evident in older bilinguals when compared to their monolingual counterparts (e.g., Bialystok et al., 2008; Hernández et al., 2010). However, as we will explain below, recent results have challenged these findings showing negligible differences between bilinguals and monolinguals in the Stroop task (Duñabeitia et al., 2014).

Evidence in favor of the so-called bilingual advantage has been also obtained using the Simon paradigm (Simon and Rudell, 1967). In this task, participants have to respond with either their left or right hand depending on one specific feature of the stimulus (e.g., the color), while ignoring other salient but apparently irrelevant features of the target (e.g., its location). The Simon task includes congruent and incongruent conditions, as a function of the match between the relevant and irrelevant features. The difference between congruent and incongruent trials (the Simon effect) has been typically found to be smaller in bilinguals than in monolinguals (Bialystok et al., 2004). Again, as with the Stroop task, this bilingual advantage has been found to be much stronger in older than in younger adults (Bialystok et al., 2004). However, as in the case of the Stroop task, recent studies have also reported negligible differences between bilinguals and monolinguals in the Simon task (see Prior and MacWhinney, 2010; Humphrey and Valian, 2012; Kousaie and Phillips, 2012; Kirk et al., 2013; Paap and Greenberg, 2013; Sawi and Paap, 2013; Gathercole et al., 2014).

Another task extensively used in the attention domain to show the bilingual advantage is the Attentional Network Test (ANT; Fan et al., 2002). This task, which is a combination of the classic flanker task (Eriksen and Eriksen, 1974) and the cueing task (Posner, 1980), measures the three independent attentional networks of Orienting, Alerting and Executive control (e.g., Fan et al., 2003). In this task, participants need to respond to the presence of an arrow on the screen, by indicating whether the arrow is pointing to the left or to the right. The critical arrow (e.g., →) can be flanked by another 2 arrows on each side, either pointing in the same direction (Congruent trials; e.g., →→→→→) or in the opposite direction (Incongruent trials; e.g., ←←→←←). Simple lines can also flank the central arrow, this way creating the Neutral condition (e.g., - - → - -). Previous to each flanker trial and after a random time period, participants can be cued about the position where the arrows are going to appear, since the arrows can appear either in the upper or in the lower part of the screen. The Cue factor can be manipulated so that participants see a valid Spatial Cue (i.e., an asterisk in a congruent cueing position), a Double Cue (i.e., one asterisk in the upper part and another one in the lower part), a Neutral Cue (an asterisk in the middle of the screen) or No Cue at all. With the combination of these 4 cue conditions (Double, Spatial, Center and No cue) and 3 flanker conditions (Congruent, Incongruent and Neutral), a measurement of the three attentional networks can be obtained. The index of the Alerting Network can be obtained by subtracting the reaction times in the Double Cue condition and the ones in the No Cue condition. Similarly, the Orienting index can be obtained by comparing the Central Cue and the Spatial Cue conditions. Finally, and possibly the most important for our purposes, the Conflict Effect, which is closely related to executive control, can be obtained by comparing the reaction times to Incongruent and Congruent trials.

In the Revised ANT task (ANT-R, Fan et al., 2009) a fifth cueing condition was created: the Invalid Spatial Cue. This was conceived as the opposite of the Valid Spatial Cue, which precedes the target stimuli in its exact same position. The Invalid Spatial Cue precedes the target arrow in the opposite part of the screen, so that an asterisk in the lower part would precede targets appearing in the upper part of the screen, and an asterisk in the upper part would precede targets appearing in the lower part. By comparing the (longer) latencies to the Invalid Cue condition to the (shorter) reaction times to the Valid Cue trials, the Validity index is obtained, considered as an index of reorienting attention.

The ANT task has been found to show a different developmental pattern for the different networks. Rueda et al. (2004), tested children from 6 to 10 years of age in an adapted version of the ANT task where the arrows were replaced with fishes to make it more child-friendly. Not surprisingly, they found that overall reaction times and error rates decreased gradually as a function of age. When the Alerting, Orienting, and Conflict networks were analyzed separately, the authors found that the developmental pattern was not parallel for these three networks. On the one hand, the Alerting network showed negligible changes between ages 6 and 10. Similarly, the Orienting network failed to show a clear-cut developmental change. In contrast, the Conflict effect showed a remarkable improvement from age 6 to age 7, remaining relatively stable after that.

Similarly to the Stroop and the Simon tasks, when the ANT task has been used to explore differences between bilinguals and monolinguals, an intriguing pattern has been found. For instance, Costa et al. (2008) tested Catalan-Spanish bilinguals and compared them to their monolingual peers. When looking at the specific attention networks, they found that monolinguals showed larger Conflict effects than bilinguals. Besides, in the Alerting network, bilingual participants showed larger benefits than monolinguals due the presence of an Alerting Cue. They also reported that bilingual participants were overall faster than their monolingual peers regardless of the Flanker and Cue type, and they showed that the overall RT differences could not be simply explained by bilinguals just being better than monolinguals at conflict resolution, given that they were also faster in congruent trials. Taken together, these results led them to abandon the hypothesis that the bilingual advantage was the consequence of bilinguals' better ability to process incongruent information, and to propose that it reflected bilinguals' enhanced monitoring abilities.

To further test this hypothesis, Costa et al. (2009) ran a version of the ANT manipulating the monitoring demands using different groups of bilingual and monolingual participants. In a first experiment they created a low-monitoring context, with 92% of the trials belonging to one condition (either Congruent or Incongruent) and 8% to the other condition, thus making the condition of the upcoming target highly predictable. In a second experiment, they created two high-monitoring contexts. In one of the contexts, each condition (i.e., Congruent and Incongruent) was represented by 50% of the trials, making it difficult to predict the condition of the individual trial. In the other context, the authors opted for a 75% congruent-25% incongruent distribution of the trials. Costa et al. found that bilingual participants were overall faster than monolinguals in the highest monitoring context (namely, 50% of the trials per condition), but did not show differences in the magnitude of the Conflict effect. Contrarily, in the low-monitoring context, both groups behaved similarly, with no differences in overall RTs or in the magnitude of the Conflict effect. In the 75–25% context a slight advantage was found in overall RTs and in the Conflict effect for bilinguals, but these effects were modest and exclusively confined to the first experimental block. Hence, the results reported by Costa et al. suggest that (1) the so-called bilingual advantage does not seem to be exclusively related to an enhancement of bilinguals' inhibitory skills (Green, 1998; Bialystok et al., 2004; Kroll et al., 2008; and see also Morales et al., 2013 for an explanation combining inhibitory and monitoring skills), and that (2) the appearance of the bilingual advantage seems to be restricted to certain experimental conditions, often failing in its replication (e.g., Prior and MacWhinney, 2010; Kousaie and Phillips, 2012; Paap and Greenberg, 2013).

Clearly at odds with these findings reported by Costa et al. (2009), a recent study by Pelham and Abrams (2014) testing young adults who were early bilinguals, late bilinguals or monolinguals in the ANT showed a significant bilingual advantage in conflict resolution. They found that monolinguals were slower than the two bilingual groups in incongruent trials, showing larger conflict effects than both late and early bilinguals (with no differences between the last two).

Although the main focus of bilingualism research using the ANT task has been the Conflict effect, given its direct relationship with executive control and its implications for the bilingual advantage based on inhibitory skills; it is worth noting that there has also been evidence of differences in the Alerting effect (Costa et al., 2008; but see Costa et al., 2009) and in the Orienting network (Colzato et al., 2008; but see Hernández et al., 2010). Clearly, it is difficult to extract a take-home-message from the bulk of evidence gathered from ANT studies with bilingual and monolingual adult samples, given the high degree of variance in the observed results.

Leaving aside the debate about critical experimental settings, tasks or contexts that lead to the appearance or vanishing of the bilingual advantage, it is worth noting that the strongest pieces of evidence supporting it come from adult research and especially from research done with elder adults. However, this bilingual advantage is more elusive in research with children and the number of discrepant studies of this type has increased in recent years. Curiously, it should be mentioned that even researchers showing differences between bilingual and monolingual adults admit that the evidence in favor of a bilingual advantage in children is certainly limited (Bialystok et al., 2010, 2012; see also Hilchey and Klein, 2011, for a review). Furthermore, it has been suggested that some factors other than the mere linguistic profile of the participants may play a very important role in the emergence of the bilingual advantage in different tasks. For instance, Morton and Harper (2007) tested a group of bilingual and monolingual children in a Simon task and they found no differences in their performance as a function of the number of languages they knew. Instead, they found a significant correlation between their socio-economic status (SES) and their performance in the task, arguing that the SES, not bilingualism, was the crucial factor in producing the effect. Hence, the number of intra-experimental and external factors that seem to have a direct impact on the appearance (and the magnitude) of the bilingual advantage is increasing, and the true nature of bilingual outperformance in executive control tasks remains unclear, casting doubts on some of the claims that have lead the field in the last decade. In this line, Paap and Greenberg (2013) recently reported that the studies which have failed to obtain a bilingual advantage should not be ignored. They noted that many of the studies showing a bilingual advantage could possibly be showing a Type I error, due to inadequately matched or very small groups, uncontrolled external factors or task-dependency effects. They concluded that the replicability and the cross-study reliability of this advantage are markedly low.

Following this line of reasoning, in a recent study, Duñabeitia et al. (2014) compared the performance of a group of more than 250 bilingual children to that of a group of very well matched monolinguals in both the classic Stroop task and the Numerical Stroop task (a variation of the classic task with minimal involvement of language). Following the claims raised, among others, by Paap and Greenberg (2013) and Morton and Harper (2007), Duñabeitia et al. carefully matched participants for age, reading and mathematical abilities, and verbal and non-verbal IQ, together with some socio-economic indicators. In a series of different analyses, Duñabeitia et al. found no signs of a difference in the performance of these two groups. These findings lead the authors to conclude that the so-called bilingual advantage in executive control tasks seems to be inexistent in children. Nonetheless, as they acknowledged, further research is needed in order to shed light on the replicability of the bilingual advantage across tasks.

These conclusions are also endorsed by a recent study by Gathercole et al. (2014), who tested a large number of Welsh children and adults in different tasks (*n* = 650 in a card sorting task, *n* = 557 in the Simon task and *n* = 354 in a grammaticality judgment task). The different groups tested included English monolinguals and bilinguals coming with different degrees of use of Welsh and English (i.e., bilinguals who only spoke Welsh at home, bilinguals who used both Welsh and English at home, and bilinguals coming from English-speaking homes). Importantly, Gathercole et al. found no evidence for a bilingual advantage. No differences were found in the switch cost or overall performance in the card sorting task. Similarly, negligible differences were found in the Simon task. The grammaticality judgment task also failed to reveal any systematic bilingual advantage.

Considering the lively debate about how bilingualism may affect performance in the ANT task, in the current study we tested a group of 360 children (180 bilinguals, 180 monolinguals) of different ages in a child-friendly version of the ANT (see Rueda et al., 2004). Similarly to the careful matching of the participants tested in the study by Duñabeitia et al. (2014), special care was taken to avoid the influence of uncontrolled factors in the data observed. Following the inconsistent results obtained in the ANT with adult participants (see Costa et al., 2009; Pelham and Abrams, 2014), the absence of a bilingual advantage in the study with children presented by Duñabeitia et al. (2014), and the results reported by Rueda et al. regarding the different development of the attention networks as a function of age, here we investigated (1) whether there is a bilingual advantage in children in any of the attention networks, and (2) whether the development of these networks is similar or different for bilingual and monolingual children.

## Methods

### Participants

Two groups of participants were recruited from different schools in Spain (*n* = 360, females = 211). The first group was made up of 180 Spanish monolingual children (females = 106) from second, third, fourth and fifth grades of elementary school and grade one from secondary school. These monolinguals were recruited from Spanish schools in places where Spanish is the only official language, and none of them had fluent knowledge of any other language than Spanish. Also, none of them corresponded to any immigrant minority and they were only exposed to Spanish at home. The second group was formed by 180 bilingual children (females = 105) from the same grades who were born and lived in the Basque Country. The Basque Country is a Spanish region where two languages, Basque and Spanish, are co-official. All these bilingual children were attending bilingual schools where both languages were used as vehicular languages. According to the legal requirements, bilingual schools in the Basque Country ensure that teachers switch from one to the other language as they switch academic subjects, making sure of a similar distribution of the languages across subjects and school time (50% in each language). This way, Basque children attending bilingual schools are exposed actively to the two languages on a daily basis during schooling. A linguistic competence questionnaire filled in by 171 of the 180 bilingual children's parents (namely, 95% of the sample) showed that bilingual participants had acquired the two languages very early in life, with overall age-of-acquisition scores of 0.58 years (*SD* = 0.77) for Spanish and of 2.23 years (*SD* = 1.07) for Basque. The parents' subjective ratings for the children's performance in Basque and Spanish were collected on a 0-to-10 scale, where 10 represented a perfect knowledge and use of the language. Children's mean proficiency scores in Spanish was 8.65 (*SD* = 1.17), and their score in Basque was 5.96 (*SD* = 1.63).

The reason for selecting samples of children instead of adult samples is twofold. First, considering the idiosyncrasy of the bilingual educational system in the Basque Country (see above), a relatively high degree of control of children's use of the two languages can be applied. Simply by checking their academic syllabus and the language in which each subject is being taught, daily exposure to both languages can be ensured. And second, considering that the most reliable pieces of evidence supporting the so-called bilingual advantage have been obtained for individuals that are not at ceiling level in their executive functions (e.g., elderly), it could be tentatively suggested that any difference between bilinguals and monolinguals should also emerge in samples of individuals who have not reached yet a fully developed attentional system (e.g., children). The different cognitive and executive skills develop progressively during childhood, and while some of them are relatively mature around age 12–13, many other executive processes are only fully developed or established during mid-adolescence or adulthood (see Anderson, 2002, for review).

In order to explore the developmental trajectory of the attention networks, we divided the sample of bilinguals and monolinguals into three evenly distributed subgroups. Monolingual and bilingual 2nd and 3rd graders were classified as Group 1, 4th and 5th graders were classified as Group 2, and 6th graders and students from the first high school grade were classified as Group 3. 120 children were included in each group, half of them (*n* = 60) corresponding to a monolingual environment and the other half corresponding to a bilingual context. Pairwise comparisons within each group showed no differences (all *p*s > 0.11) between bilinguals and monolinguals in age, gender, overall reading and arithmetic skills (as assessed by their teachers on a 1-to-5 Likert scale), verbal, non-verbal and composed IQ [obtained from the Spanish version of the Kaufman Brief Intelligence Test (1990), K-BIT], income at home (classified according to the following categories: >3000€/month, category 1; 2001–3000€, category 2; 1601–2000€, category 3; 1201–1600€, category 4; 750–1200€, category 5 and <750€ category 6), number of years of formal education of the parents, and parental work status (including three possible categories: neither works, only one of them works, both of them work). Furthermore, we made sure that none of the participants had any specific developmental, psychological, psychiatric, or educational disorder, deficit, or special need by including a series of questions in this regard in the questionnaires completed by parents and teachers. Besides, none of the children had repeated any academic year and no child with scores below the 20th centile in verbal, non-verbal, and combined IQ tests was included in the sample. Hence, the two groups were carefully matched in many socio-economic and cognitive measures (see Table 1 for detailed comparisons).

**Table 1:** **Characteristics of the samples tested in the experiment**.

**Age group**	**Language group**	**Age**		**Reading scores**		**Math scores**		**Verbal IQ**		**Non-verbal IQ**		**General IQ**		**Incomes**		**Parents' education**		**Parents'work situation**	
		**(in years)**		**(1–5)**		**(1–5)**		**(centiles)**		**(centiles)**		**(centiles)**		**(category)**		**(years)**		**(category)**	
		**Mean**	***SD***	**Mean**	***SD***	**Mean**	***SD***	**Mean**	***SD***	**Mean**	***SD***	**Mean**	***SD***	**Mean**	***SD***	**Mean**	***SD***	**Mean**	***SD***
Group 1	Bilinguals	7.57	0.59	4.53	1.17	4.52	0.93	77.18	14.58	63.00	22.31	68.82	17.88	1.98	1.07	14.30	2.49	1.90	0.35
Primary 2nd and 3rd	Monolinguals	7.55	0.53	4.57	0.98	4.57	0.87	79.28	15.76	60.85	22.18	69.73	19.74	2.15	0.99	13.88	2.76	1.90	0.35
	*p* value	0.88		0.84		0.72		0.31		0.48		0.70		0.25		0.29		1.00	
Group 2	Bilinguals	9.53	0.57	4.75	0.95	4.87	0.89	63.72	18.62	66.13	18.43	62.30	17.56	1.77	0.96	14.59	2.16	2.00	0.00
Primary 4th and 5th	Monolinguals	9.50	0.60	4.78	0.83	4.82	0.87	65.32	19.12	66.53	17.81	63.32	17.13	1.88	0.94	14.44	2.39	2.00	0.00
	*p* value	0.78		0.84		0.75		0.65		0.90		0.76		0.55		0.71		1.00	
Group 3	Bilinguals	11.43	0.65	4.57	1.06	4.42	0.91	56.93	18.23	68.03	17.90	59.52	17.64	1.48	0.68	14.62	2.30	1.92	0.28
Primary 6th and Secondary 1st	Monolinguals	11.47	0.54	4.58	0.91	4.63	0.84	61.20	17.73	63.10	19.78	59.37	19.28	1.65	0.66	14.07	2.34	1.95	0.22
	*p* value	0.73		0.92		0.13		0.12		0.11		0.96		0.17		0.18		0.42	
Total	Bilinguals	9.51	1.69	4.62	1.06	4.60	0.93	65.94	19.11	65.72	19.64	63.54	18.02	1.74	0.93	14.50	2.31	1.94	0.26
	Monolinguals	9.51	1.70	4.64	0.91	4.67	0.86	68.60	19.14	63.49	20.03	64.14	19.14	1.89	0.89	14.13	2.50	1.95	0.24
	*p* value	0.93		0.79		0.42		0.16		0.31		0.77		0.13		0.12		0.66	

### Design

In this version of the child Attention Network Test (ANT) two within-subject factors were manipulated, Cue type (Double Cue, Valid Cue, Invalid Cue, Neutral Cue and No Cue) and Flanker type (Incongruent, Congruent), leading to a total of 10 conditions. As already explained in the Introduction, Fan et al. (2009) suggested that the inclusion of an index of validity within the cueing conditions provides an additional measure of the ability to reorient attention. Hence, valid and invalid cues were included in the current design too. The cueing manipulations were created by presenting (or not) an asterisk on the screen prior to the presentation of the target strings. These cues could be presented at the same position of the upcoming target (Valid condition), or in the opposite position (Invalid condition). In order to create the Double Cue condition, two asterisks were presented at the same time above and below the center of the screen. The Neutral Cueing condition was created by presenting the asterisk at the center of the screen, and the No Cue condition was created by not providing any visual cue. Regarding the flanker manipulation, the target was a left- or right-pointing yellow fish (1.6°), presented above or below the fixation cross. This central fish was flanked on both sides by two fishes pointing either in the same direction (Congruent trials), or in the opposite direction (Incongruent trials). The distance between the fishes was 0.21°. The target and flankers subtended 8.84° and were presented 1° above and below the fixation cross over a blue-green background. For detailed description of the stimuli and procedure, see Rueda et al. (2004).

### Procedure

All the stimuli were presented on a computer screen. Each trial began with a fixation cross (1° of visual angle) with a random duration between 400 and 1600 ms. Then a cue (an asterisk) could appear in any of its variants (see below) for 150 ms. Next, a centered fixation cross appeared on the screen for 450 ms, immediately followed by the target and flanker stimuli. The target string stayed on the screen until a response was given or for a maximum of 1700 ms. After each trial, feedback was provided.

A session of the ANT consisted in a total of 288 trials. Each trial represented one of the 10 conditions mentioned above (Cue type × Flanker type). To keep the high-monitoring demanding context, 50% of the trials belonged to the Congruent condition and the other 50% to the Incongruent condition. Regarding each cueing condition, there were 72 Double Cue, 48 Valid, 48 Invalid, 48 Neutral Cue and 72 No Cue trials. Participants were seated at a distance of about 55 cm from the screen and they were instructed with a series of practice trials to indicate the direction of the central fishes of the strings, pressing the “L” key in the keyboard for right responses or the “S” key for left responses. Both accuracy and reaction times were recorded in each experimental trial.

### Data analysis

Reaction times below 200 ms (only representing 0.12% of the data) were excluded. Reaction time data was trimmed by using the classic 2.5SD criterion, resulting in the exclusion of the 2.49% of the data, and RTs associated with erroneous responses were not included in the latency analyses. Before focusing on the individual indices for each attention network, all the conditions were analyzed in a general ANOVA including Cue Type (No Cue, Valid Cue, Invalid Cue, Double Cue and Neutral Cue) and Flanker Type (Congruent and Incongruent) as within-participant factors, and Language (Bilinguals and Monolinguals) and Group (First, Second and Third group) as between-participants factors. In subsequent analyses we looked at the different attention networks by measuring the following indexes: the difference between Congruent and Incongruent trials as a reflection of executive control (Conflict effect), the differences between the Double Cue and the No Cue conditions for the alerting network (Alerting effect), the orienting network as measured by the difference between the trials with a Neutral Cue and trials with a Valid Cue (Orienting effect), and finally the difference between the trials with a Valid Cue vs. the trials with an Invalid Cue as markers of the Validity effect. Detailed information about the RT and error data is presented in Table 2.

**Table 2:** **Reaction times and error rates to each condition**.

	**Conditions**															
	**Double Cue**		**Neutral Cue**		**Valid Cue**		**Invalid Cue**		**No Cue**		**Congruent**		**Incongruent**		**Total**	
	**Mean**	***SD***	**Mean**	***SD***	**Mean**	***SD***	**Mean**	***SD***	**Mean**	***SD***	**Mean**	***SD***	**Mean**	***SD***	**Mean**	***SD***
**REACTION TIMES**																
Bilinguals	690.30	110.13	705.87	112.55	672.67	107.13	724.39	103.20	714.49	108.34	670.88	104.01	732.21	109.47	701.55	105.75
Monolinguals	676.12	101.31	692.86	111.44	659.59	106.71	711.09	108.94	703.99	105.34	659.41	103.97	718.05	106.95	688.73	104.48
**ERROR RATES**																
Bilinguals	4.92	5.40	4.92	5.41	4.56	5.30	5.91	6.30	5.69	5.61	3.45	4.58	6.95	5.90	5.20	4.91
Monolinguals	4.58	5.64	4.99	5.72	4.20	5.84	5.60	6.43	5.02	5.68	3.18	4.78	6.57	6.30	4.88	5.28

## Results

### General analyses

In the RT analysis, we found significant main effects of Flanker Type [*F*_(1, 354)_ = 1624.68, *MSE* = 1993.35, *p* < 0.01], Cue Type [*F*_(4, 1416)_ = 237.19, *MSE* = 1298.75, *p* < 0.01] and Group [*F*_(2, 354)_ = 120.07, *MSE* = 66486.08, *p* < 0.01]. In contrast, the main effect of Language was not significant [*F*_(1, 354)_ = 2.22, *MSE* = 66486.08, *p* > 0.13]. The 2-way interaction between Flanker Type and Group was significant [*F*_(2, 354)_ = 12.5, *MSE* = 1993.35, *p* < 0.01], and the same was true for the interaction between Flanker Type and Cue Type [*F*_(4, 1416)_ = 24.12, *MSE* = 893.76, *p* < 0.01]. None of the other interactions was significant.

In error rate analysis, both Language groups performed similarly (*F* < 1). The main effects of Flanker Type [*F*_(1, 354)_ = 303.20, *MSE* = 35.25, *p* < 0.01], Cue Type [*F*_(4, 1416)_ = 11.52, *MSE* = 17.61, *p* < 0.01], and Group [*F*_(2, 354)_ = 43.53, *MSE* = 210.73, *p* < 0.01] were significant. The only significant interactions found were the Flanker Type ^*^ Group interaction [*F*_(2, 354)_ = 6.85, *MSE* = 35.25, *p* < 0.01], and the Flanker Type ^*^ Cue Type interaction [*F*_(4, 1416)_ = 90.32, *MSE* = 17.44, *p* < 0.01].

Thus it is important to notice that none of the interactions with Language were significant, showing that the same effects hold for bilinguals and monolinguals.

### The three attentional networks

**Figure 1:**
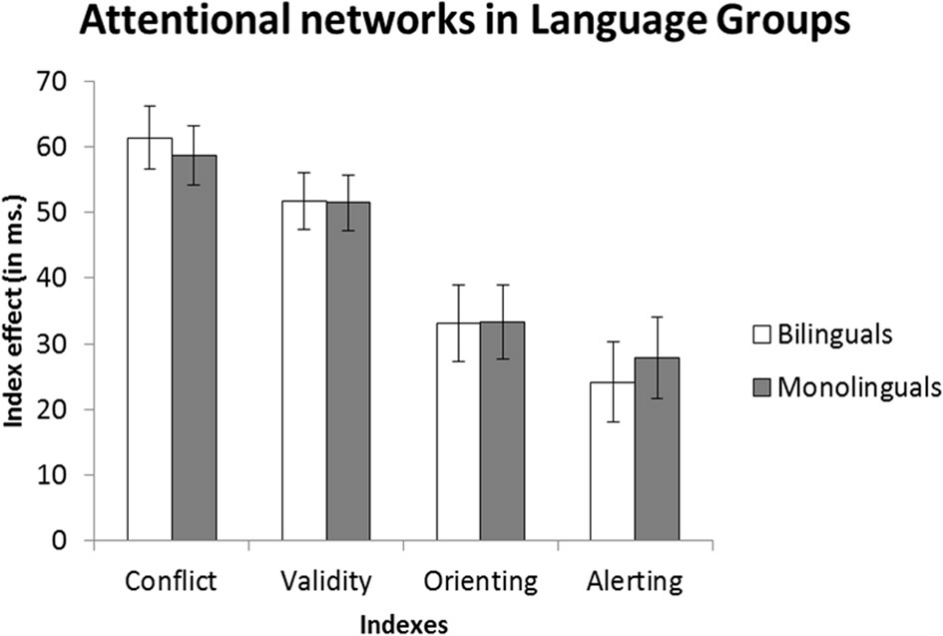
**Comparison of indexes across Language Groups**.

**Figure 2:**
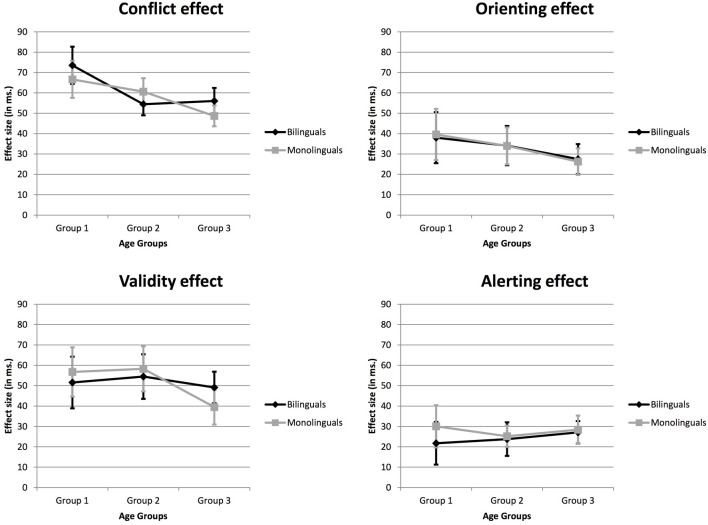
**The four indexes, representing the attentional networks, across age groups and language groups**.

Considering the reliable Flanker Type ^*^ Cue Type interactions, and following preceding research, we explored each of the effects mentioned above individually (i.e., Conflict, Alerting, Orienting and Validity), and the manner in which the between-participants factors Group and Language could modulate them (see Table 3 and Figure 1 for comparisons between Language groups; and see Table 4 and Figure 2 for a detailed comparison between Language Groups in each Age Group).

**Table 3:** **Attentional networks, measured as the difference in reaction times and error rates**.

	**Attentional networks**							
	**Conflict index**		**Orienting index**		**Alerting index**		**Validity index**	
	**Mean**	***SD***	**Mean**	***SD***	**Mean**	***SD***	**Mean**	***SD***
**REACTION TIMES**								
Bilinguals	61.34	29.51	33.20	39.85	24.19	32.82	51.72	41.95
Monolinguals	58.64	28.94	33.27	38.50	27.87	30.76	51.50	42.78
**ERROR RATES**								
Bilinguals	3.50	3.91	0.36	4.22	0.76	4.00	1.35	4.51
Monolinguals	3.39	3.71	0.79	3.77	0.43	4.09	1.40	4.28

**Table 4:** **Latency differences in attentional networks in each age group**.

	**Conflict effect**				**Orienting effect**				**Alerting effect**				**Validity effect**			
**Age Group**	**Bilinguals**		**Monolinguals**		**Bilinguals**		**Monolinguals**		**Bilinguals**		**Monolinguals**		**Bilinguals**		**Monolinguals**	
Group 1	73.53	(36.21)	66.63	(35.81)	38.02	(49.51)	39.59	(49.61)	21.71	(41.38)	30.06	(41.01)	51.56	(50.14)	56.76	(47.72)
Group 2	54.44	(21.51)	60.60	(26.15)	34.12	(38.12)	33.95	(35.92)	23.77	(32.63)	25.18	(20.58)	54.47	(43.28)	58.27	(43.66)
Group 3	56.04	(25.29)	48.69	(20.12)	27.47	(29.25)	26.26	(25.62)	27.09	(21.82)	28.37	(27.44)	49.14	(30.63)	39.49	(33.88)

Means and SD (in parenthesis) are displayed.

#### Executive network: the conflict effect

In the RT analysis, the Conflict effect as measured by the factor Condition (Congruent vs. Incongruent trials) was significant [*F*_(1, 354)_ = 1624.68, *MSE* = 398.67, *p* < 0.01], as well as the main effect of Group [*F*_(2, 354)_ = 120.07, *MSE* = 13297.22, *p* < 0.01] and the interaction between them [*F*_(2, 354)_ = 12.50, *MSE* = 398.67, *p* < 0.01]. It took longer for participants to respond to the Incongruent trials as compared to the Congruent ones, and participant speed of response increased as a function of age (see below). Importantly, the main effect of Language was not significant [*F*_(1, 354)_ = 2.22, *MSE* = 13297.22, *p* > 0.13], and it did not interact with Condition (*F* < 1) or with Group (*F* < 1). The 3-way Language^*^Condition^*^Group interaction was not significant [*F*_(2, 354)_ = 2.22, *MSE* = 398.67, *p* > 0.11]. Hence, we can conclude that monolinguals and bilinguals showed highly similar Conflict effects.

In order to explore the origin of the significant Condition^*^Group interaction, follow-up contrasts were run collapsing the data across linguistic profiles. Pairwise contrasts showed that the differences in the responses to the two types of flankers (Congruent, Incongruent) decreased with age. Thus, when comparing the Conflict effect in each Group, we observed that the first group showed the largest Conflict effect (average of 70 ms), and that this effect progressively diminished with age (Group 2 = 57 ms; Group 3 = 52 ms). Pairwise tests showed that the effect was significantly larger for Group 1 than for Group 2 and Group 3 [Group 1 vs. Group 2: *t*_(238)_ = 3.18, *p* < 0.01; Group 1 vs. Group 3: *t*_(238)_ = 4.54, *p* < 0.01], while the difference was not significant between Groups 2 and 3 [*t*_(238)_ = 1.70, *p* < 0.1].

In error rate analysis only the main effects of Condition [*F*_(1, 354)_ = 303.20, *MSE* = 7.05, *p* < 0.01] and Group [*F*_(2, 354)_ = 43.53, *MSE* = 42.15, *p* < 0.01] were significant. The only significant interaction was found between Condition and Group [*F*_(1, 354)_ = 6.85, *MSE* = 7.05, *p* < 0.01]. Replicating the RT data, the error data showed a clear Conflict effect, with higher error rates in incongruent than in congruent conditions and a modulation of the percentages of errors as a function of age (i.e., overall error rates diminished as a function of age). Given the significant interaction, we can conclude that the magnitude of the Conflict effect decreased as a function of age. Importantly, the Language effect and the interactions between this and the other factors were negligible (all *F*s < 1 and all *p*s > 0.5).

#### Alerting network: the alerting effect

When considering the differences in RTs between the Double Cue and the No Cue conditions, only the main effects of Condition [*F*_(1, 354)_ = 239.44, *MSE* = 509.37, *p* < 0.01] and Group [*F*_(2, 354)_ = 118.55, *MSE* = 13364.56, *p* < 0.01] were significant. The Language effect was not significant [*F*_(1, 354)_ = 2.05, *MSE* = 13364.56, *p* > 0.15]. None of the interactions were significant (*F*s < 1.20, *p*s > 0.27]. Hence, participants responded faster to Double Cue trials than to No Cue trials and they became overall faster as their age increased but the difference between the cueing conditions did not differ across ages or across language profiles.

In the error rate analysis, the only significant effects corresponded to the factors Condition [*F*_(1, 354)_ = 7.81, *MSE* = 8.25, *p* < 0.01] and Group [*F*_(2, 354)_ = 41.25, *MSE* = 44.43, *p* < 0.01], showing that participants made more errors in No Cue trials than in Double Cue trials and that the number of errors decreased as a function of age. No other effects or interactions were significant (all *F*s < 1.1 and all *p*s > 0.3).

#### Orienting network: the orienting effect

The Orienting effect (i.e., Valid Cue vs. Neutral Cue) was significant [*F*_(1, 354)_ = 260.30, *MSE* = 763.89, *p* < 0.01], as was the main effect of Group [*F*_(2, 354)_ = 109.45, *MSE* = 14488.40, *p* < 0.01]. Responses to trials with a Valid Cue were faster than responses to trials with a Neutral Cue and averages RTs decreased as a function of age. In contrast, the main effect of Language was not significant [*F*_(1, 354)_ = 2.12, *MSE* = 14488.40, *p* > 0.14], and none of the interactions involving the factor Language was significant (all *F*s. < 1). A marginal interaction between Condition and Group was found [*F*_(2, 354)_ = 2.84, *MSE* = 763.89, *p* < 0.07], suggesting that the magnitude of the Orienting effect decreased with age. Follow-up pairwise contrasts showed similar Orienting effects for Groups 1 and 2 (39 and 34 ms, respectively; *t* < 1), and a significantly smaller effect for Group 3 [27 ms; Group 1 vs. Group 3: *t*_(238)_ = 2.32, *p* < 0.03; Group 2 vs. Group 3: *t*_(238)_ = 1.71, *p* < 0.09].

In the error rate analysis, the only significant effects found were in Condition [*F*_(1, 354)_ = 7.33, *MSE* = 8.06, *p* < 0.01], showing more errors in the Neutral Cue condition than in the Valid Cue condition, and Group [*F*_(2, 354)_ = 34.74, *MSE* = 45.66, *p* < 0.01], showing a decrease in the amount of errors as a function of age. No other effects or interactions were significant (all *F*s < 1.1 and all *p*s > 0.3).

#### Reorienting: the validity effect

The difference between trials with a Valid Cue and trials with an Invalid Cue were significant in the RT analysis [main Condition effect: *F*_(1, 354)_ = 539.92, *MSE* = 888.06, *p* < 0.01], and the Group effect was also significant [*F*_(2, 354)_ = 117.92, *MSE* = 13211.03, *p* < 0.01]. Invalid Cues produced longer response times than Valid Cues, and the overall response times decreased as a function of age. These two effects marginally interacted with each other [*F*_(2, 354)_ = 2.78, *MSE* = 888.06, *p* < 0.07], suggesting that the magnitude of the Validity effect decreased with age. Follow-up *t*-tests showed that the magnitude of the Validity effect was similar for Groups 1 and 2 (54 and 56 ms, respectively; *t* < 1), and that the effect was smaller for Group 3 (44 ms) than for Group 2 [*t*_(238)_ = 2.44, *p* < 0.02] and, although marginally significant, than for Group 1 [*t*_(238)_ = 1.84, *p* < 0.07]. Critically, the main effect of Language was not significant [*F*_(1, 354)_ = 2.37, *MSE* = 13211.03, *p* > 0.12], and none of the interactions involving the Language factor were significant either (all *F*s < 1.15 and *p*s > 0.32).

Parallel findings were also observed in the error rate analysis, showing significant Condition [*F*_(1, 354)_ = 35.60, *MSE* = 9.59, *p* < 0.01] and Group effects [*F*_(2, 354)_ = 37.15, *MSE* = 51.80, *p* < 0.01], together with a marginal interaction between these two factors [*F*_(2, 354)_ = 3.03, *MSE* = 9.59, *p* < 0.06]. Again, no other effects or interactions were significant (all *F*s < 1 and all *p*s > 0.5).

### Bayesian null hypothesis testing

Given that classical hypothesis testing does not allow for accepting the null hypothesis, we tested the critical differences of interest following a Bayesian approach (see Rouder et al., 2009, among others). For each index (Conflict, Validity, Orienting and Alerting), we used a Bayes factor (BF) approach to compare a model that assumed no differences between bilinguals and monolinguals (H0) against a model that assumed that bilinguals perform differently from monolinguals (H1). With this test, the null hypothesis is accepted if the resulting BF is below 0.3, and the alternative hypothesis is accepted if it is above 3 (see Kruschke, 2011, Figure 3 in page 6). When comparing bilinguals and monolinguals' Conflict effects, results favored the acceptance of the null model (*BF* < 0.18). The other three attentional networks responded similarly, all of them being better explained by a null model as compared to the alternative (*BF* < 0.12 for the Orienting effect, *BF* < 0.21 for the Alerting effect, and *BF* < 0.13 for the Validity effect). These results suggest support for the hypothesis of no difference.

**Figure 3:**
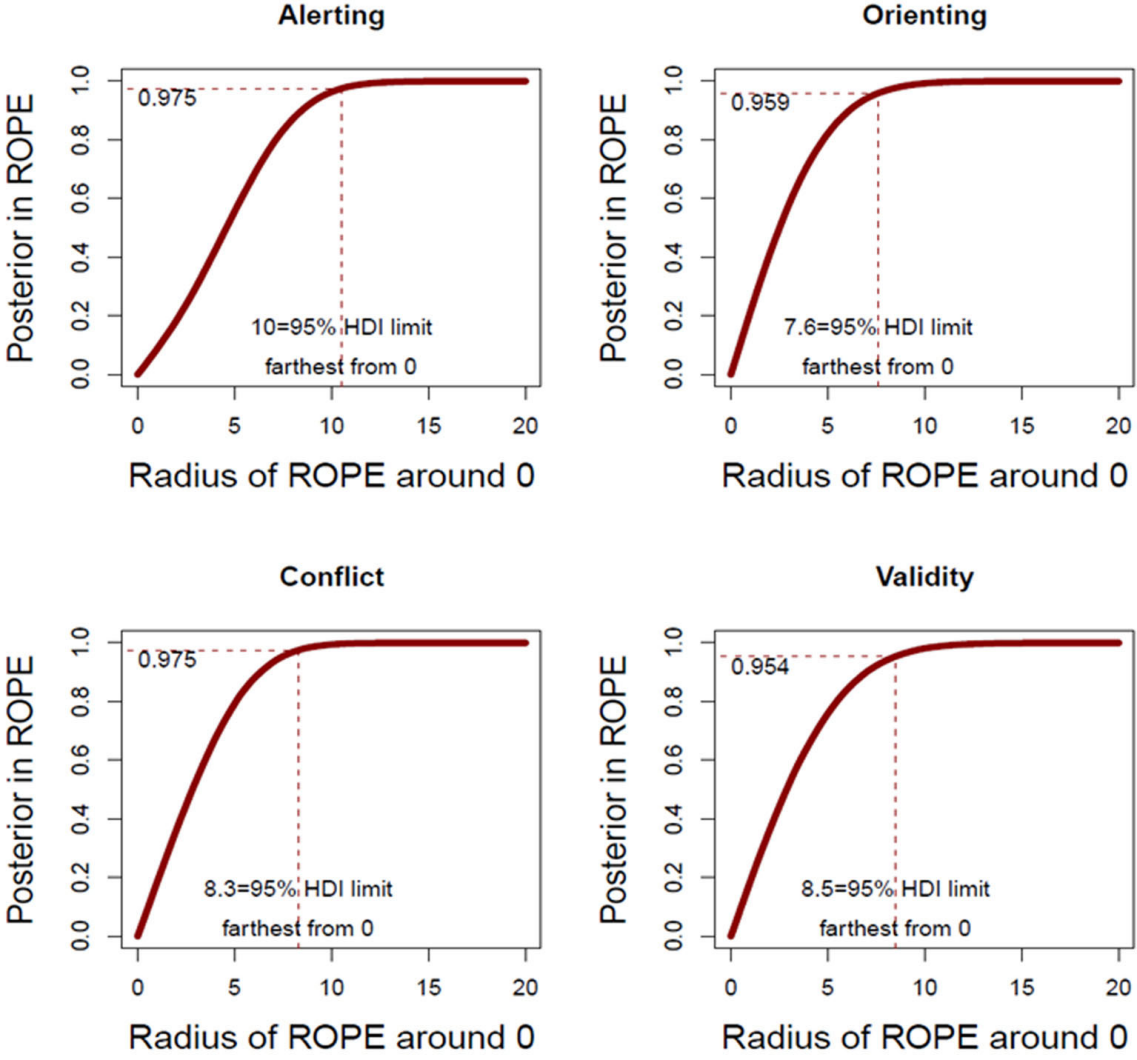
**Proportion of the posterior distribution falling within the ROPE as a function of ROPE width**. X axis shows how far the ROPE limit is from 0 value (no differences). Y axis reflects the proportion of the posterior distribution that falls inside the ROPE. Dotted line shows the proportion at the right edge of the highest posterior density interval (HDI).

We further explored the reliability of the current lack of differences using Bayesian Parameter Estimation by testing the degree of confidence of the null value with the Region of Practical Equivalence (i.e., ROPE; see Kruschke, 2013, for details). Following this approach, a ROPE comprising the range of values assumed to be statistically equal to the null value (i.e., how much of a difference is accepted to be considered equal to no differences at all) is determined by previous findings in the field. If at least 95% of the posterior distribution (i.e., the prior distribution updated by the distribution of the current data) falls within this ROPE, the null hypothesis should be accepted. In contrast, if 95% of the posterior distribution falls outside the ROPE, then the alternative hypothesis should be accepted. The ROPE width would ideally be taken from preceding similar studies, but in this case there is not a consensus in the literature about the smallest meaningful difference. Therefore, we calculated the proportion of the posterior falling within the ROPE boundaries for a range of ROPE limits from 0 to 20 ms in each index (Conflict, Alerting, Orienting and Validity). This approach allows us to calculate the range of values surrounding 0 that should be accepted as equivalent to no-differences (i.e., the ROPE) to accept the null hypothesis. As seen in Figure 3, in order to get the 95% or more of our posterior distributions within the ROPEs, the radii of the ROPEs need to be set to values ranging from 7.6 to 10 ms (10 ms for Alerting, 7.6 for Orienting, 8.3 for Conflict and 8.5 for Validity). In essence, this means that, if we accept differences between 7.6 and 10 ms as equivalent to no differences at all, and given that then the majority of the distribution of the differences falls below these limits, we take this as support for the null hypothesis(^1^). Considering that the differences found between bilinguals and monolinguals in the four indices are far below these cutoff points (4 ms for Alerting, 0 ms for Orienting, 3 ms for Conflict and 0 ms for Validity), considering also that reliable differences in RTs of 10 ms in studies of children are rarely reported (note also that even in adult differences of 10 ms in the conflict effect between bilinguals and monolinguals may result in a non-significant effect (see Costa et al., 2009), we believe the data support the null hypothesis (no differences between bilinguals and monolinguals).

## General discussion

The aim of this study was to investigate whether bilingual children exhibit an advantage as compared to their monolingual peers in the ANT task, which has been typically considered the paradigm best suited to explore the different attention networks. As described in the Introduction, different explanations have been given for the so-called bilingual advantage (see Green and Abulatebi, 2013; Kroll and Bialystok, 2013); but all of them coincide in suggesting that the continuous use and control of (and switching between) two languages provides bilinguals with a set of enhanced attention skills that ultimately leads to the emergence of differences between monolinguals and bilinguals in different non-linguistic tasks closely associated with executive control. In light of some recent studies failing to replicate the bilingual advantage with different populations (e.g., Paap and Greenberg, 2013; Duñabeitia et al., 2014; Gathercole et al., 2014), and considering the existing debate between researchers suggesting that bilinguals outperform monolinguals in the ANT task (e.g., Kapa and Colombo, 2013; Pelham and Abrams, 2014) and those suggesting that the bilingual advantage in this task is restricted to certain conditions and designs (e.g., Costa et al., 2009), we investigated whether a large sample of bilingual children would exhibit better performance in this task than a group of carefully matched monolingual children. Our results unambiguously demonstrated that the so-called bilingual advantage could not be replicated in the ANT when a sufficiently large and well-matched group of bilingual and monolingual children were tested.

Our results add to a growing body of evidence showing that most forms of bilingual advantage in tasks exploring attention skills may well be the result of uncontrolled factors (e.g., Morton and Harper, 2007; Paap and Greenberg, 2013; see also Paap and Liu, 2014, and Paap, submitted, for review) or specific conditions associated with the design and procedure (e.g., Costa et al., 2009). Also, together with the results provided by Duñabeitia et al. (2014) from a large-scale study testing monolingual and bilingual children in two different versions of the Stroop task and by Gathercole et al. (2014), who tested a large number of Welsh-English bilinguals and English monolinguals in different tasks, these results demonstrate the clear similarity between monolingual and bilingual children in their performance in tasks with high executive control demands.

We argue that if the so-called bilingual advantage were a consequence of bilinguals' enhanced inhibitory skills, a reduced Conflict effect should have been found for the bilingual group (i.e., smaller differences between Incongruent and Congruent trials for bilinguals than for monolinguals). This was not the case, and participants performed in a highly similar fashion in these two conditions regardless of their linguistic profile. On the other hand, if the previously reported bilingual advantage were the result of bilinguals' enhanced monitoring skills, one would have expected an overall difference between groups in the RTs and/or in the error rates (e.g., Costa et al., 2009; see also Wu and Thierry, 2013), but again we did not find any supporting data for this claim (see also Duñabeitia et al., 2014, for similar results).

It is worth mentioning that the lack of a bilingual advantage in this study cannot be ascribed to a general lack of sensitivity of our design to the specific attention network(s) that may underlie such a difference between bilinguals and monolinguals. Replicating preceding evidence from the monolingual domain, we have shown that bilingual and monolingual children exhibited longer latencies and higher error rates for Incongruent trials than for Congruent trials (namely, a significant Conflict effect). Similarly, a better performance of both groups was found in the Double Cue trials as compared to the No Cue trials (namely, a significant Alerting effect). Also, participants' responses to the Valid Cue trials were faster and more accurate than their responses to Central Cue (i.e., a significant Orienting effect). Finally, participants showed longer RTs and higher error rates in trials involving an Invalid Cue than in trials with a Valid Cue (i.e., a significant Validity effect). Hence, considering that the current results fully replicate the indices observed in preceding studies with the ANT task (e.g., Fan and Posner, 2004; Fan et al., 2005; Wang and Fan, 2007; Ishigami and Klein, 2010; Yin et al., 2012; Mackie et al., 2013 among many others), it is hardly possible that potential differences between bilinguals and monolinguals were masked due to a lack of statistical power of the current study (see also the magnitude of the *F*-values at this regard). Furthermore, from a developmental point of view, the current study has replicated and extended the findings observed by Rueda et al. (2004) in a similar study testing a smaller group of monolingual children. The same developmental trend observed in that study can be seen here, suggesting that the Conflict effect (hence, the executive network) is the attentional index that is most sensitive to a developmental change, greatly diminishing as a function of age. On the other hand, we see more modest changes in the Validity and Orienting effects (note that the interactions were marginally significant in spite of the sample size), and no significant changes in the Alerting effect as a consequence of age.

In a nutshell, and in spite of the statistical power of the current study, no significant differences between bilingual and monolingual children emerged in their performance in the ANT task. Furthermore, when taking the Bayesian approach to test the null hypothesis against the alternative, the null appears as the strongest candidate. When the analysis was based on the ROPE approach, we also found support for the null hypothesis. In this analysis we found limits for the difference between groups that were in fact larger than previously reported differences in adults.

Certainly, we want to avoid generalizing the observed lack of bilingual advantage to other age groups, and as already discussed in Duñabeitia et al. (2014), our claims are exclusively endorsing the conclusion that the so-called bilingual advantage in tasks focusing on participants' attention skills is inexistent, or at best, extremely inconsistent and elusive. As discussed in the Introduction, both behavioral and neuroimaging evidence (see, among many others, Luk et al., 2010; Gold et al., 2013) suggest some form of bilingual advantage in similar tasks with adult samples. Hence, as mentioned by Kroll and Bialystok (2013), the existence of a bilingual advantage in adulthood cannot be ignored, even though the degree to which those findings can be generalized to all adult bilingual samples is limited (see Paap and Greenberg, 2013, among others). It should be considered that the so-called bilingual advantage may emerge as a consequence of lifelong bilingualism mainly in later stages of life (e.g., the elderly).

Leaving aside the debate about the stability of the bilingual advantage in attention-related skills in adulthood, what the current results highlight is that the differences observed during young and old adulthood between monolinguals and bilinguals are not observed during childhood. This, together with recent evidence showing larger differences in older than in younger participants (e.g., Gold et al., 2013), suggests a highly variable nature of the so-called bilingual advantage, which seems to be strongly dependent on a number of specific factors, among which the age of the samples should be carefully considered in future studies.

### Conflict of interest statement

The authors declare that the research was conducted in the absence of any commercial or financial relationships that could be construed as a potential conflict of interest.

## Footnotes

^1^It should be noted that this does not imply that any between-group difference larger than 10 ms would significantly allow us to accept the alternative hypothesis. For this to be the case, at least 95% of the posterior distribution should lay outside the ROPE, and this would necessarily imply a much larger difference.
